# Prevalence of Consanguineous Marriage among Saudi Citizens of Albaha, a Cross-Sectional Study

**DOI:** 10.3390/ijerph20043767

**Published:** 2023-02-20

**Authors:** Mohammad A. Albanghali

**Affiliations:** Department of Public Health, Faculty of Applied Medical Sciences, Albaha University, Albaha 65779, Saudi Arabia; mohammad.aref@bu.edu.sa

**Keywords:** relative, marriage, inbreeding, consanguinity, endogamous, Albaha, Saudi Arabia

## Abstract

Consanguineous marriage (CM) is a prevalent kind of relationship in Muslim and Arab countries, and this type of relationship is linked to several health risks. This study was conducted to determine the prevalence of (CM), its associated hereditary diseases, and health-related issues among Saudi citizens in Albaha. This cross-sectional study was conducted between March 2021 to April 2021. Saudi citizens in Albaha who were aged ≥ 18 years and willing to participate were eligible for the study. A total of 1010 participants were included in this study. In total, 757 participants were married, widowed, or divorced. CM partnerships comprised 40% (*N* = 302) of the marriages among participants, of which first- and second-cousin marriages comprised 72% and 28%, respectively. The prevalence of CM among the participants’ parents was lower than that among the participants (31% versus 40%, respectively). Children of participants in a CM were more likely to have cardiovascular diseases (*p* < 0.001), blood diseases (anaemia, thalassemia) (*p* < 0.001), cancer (*p* = 0.046), hearing loss and speech disorder (*p* = 0.003), and ophthalmic diseases (*p* = 0.037). Albaha showed a high percentage of consanguinity. An educational program must be established to enhance the population’s knowledge of the consequences of CM. The current national premarital screening program should be extended to involve more screening tests for common hereditary diseases that result from CM.

## 1. Introduction

Consanguineous marriage (CM), or relative marriage, is defined as a relationship between two blood-related partners who are second cousins or more closely related. CM is a worldwide practice; however, its prevalence is associated with different factors, such as ethnicity and religion. Approximately 20% of the world’s population is estimated to be involved in a relationship with biological relatives; intrafamilial unions are a very common mode of relationship in North African, Middle Eastern, and West Asian countries [1]. In Western and European countries, the prevalence of CM does not exceed 0.5%, whereas, in India, the prevalence is 9.9%. In Arab Gulf countries and Pakistan, where the majority of inhabitants are Muslims, the prevalence of CM ranges between 40% and 60% [1,2,3,4,5,6,7,8,9,10]. In Saudi Arabia, although the prevalence of CM ranges between 42% and 67%, different cities vary in the estimated prevalence. In cities such as Mecca, Madinah, and Riyadh, the prevalence ranges between 40% and 67%, whereas in Albaha, in 2005 the prevalence was 42%, which is the lowest in comparison to other cities in Saudi Arabia [4,6].

Several genetic disorders and health-related issues have been reported as being linked to CM in the literature, and a higher risk of noncommunicable diseases (NCDs) is associated with offspring from CMs. Evidence indicates that blood diseases (anaemia and thalassaemia); cardiovascular diseases (CVD); congenital heart diseases; breast, colorectal, prostate, and lung cancers; haemoglobinopathies; intellectual disabilities; congenital glaucoma; ciliopathies; disorders related to inborn errors of metabolism; retinal dystrophies; hearing loss; and primary microcephaly are associated with CM [11,12]. The occurrence of NCDs with a high incidence rate among a population affects both public health and the healthcare system by affecting individuals’ productivity and increasing their absenteeism due to the disease itself and/or its consequences, such as emotional wellbeing or increasing the caregiver burden. Studies indicate that more than one-third of the healthcare system’s resources is spent on patients with genetic, chromosomal, or congenital disorders [13,14,15,16,17]. Consequences resulting from the increase in the number of individuals with lifetime health issues contradict the public health recommendation (No. 1) included in the letter issued by the Saudi Health Council (Ref. 32665-1, 15 September 2022), and the goals of *Saudi Vision 2030* (a strategic plan; 2.1.3), aimed at promoting preventive health risks. Therefore, urgent action must be taken for the early detection of individuals who are affected by, or carry, genetic disorders that may lead to increased risks for NCDs among future generations.

Estimating the prevalence of CM among a specific population and the incidence of CM-related health issues would significantly improve our understanding of trends in CMs over time. In addition, it would provide decision makers with reliable knowledge for the efficient planning of public health programs. Therefore, this study was designed to determine the prevalence of CM, its associated hereditary diseases, and health-related issues among Saudi citizens in Albaha.

## 2. Materials and Methods

This cross-sectional study was conducted over 45 days, from 15 March 2021 to 29 April 2021. A self-administered, online-based questionnaire was developed and distributed to collect data from the targeted population using the snowball sampling technique.

### 2.1. Study Criteria

Saudi citizens in Albaha who were aged ≥18 years and willing to participate were eligible for the study. Based on the estimated number of people living in the Albaha region in 2010, published by the General Authority for Statistics, Saudi Arabia (*N* = 411,888), the sample size required for the study was estimated as being 384 by adjusting the confidence interval to 95% and the margin of error to 5% [18].

### 2.2. Survey Tool

A self-administered questionnaire was developed to achieve the goals of this study, and it consisted of three sections. The first section collected sociodemographic information and included questions on age, sex, education level, work status, monthly income, marital status, and province of residence. The second section included two questions on the nature of the relationship between participants and their spouses and the nature of the relationship between the participants’ parents. All participants were given three choices to answer these questions: no relation, first cousin, and second cousin. The third section of the questionnaire included a set of 17 questions that screened for hereditary diseases and health-related issues among participants, including blood pressure, diabetes, CVDs, blood diseases (anaemia and thalassaemia), obesity, cancer, Down syndrome, chronic gastrointestinal disorders, mental disorders (epilepsy and depression), hearing loss and speech disorders, rheumatoid arthritis, ophthalmic diseases, cleft lip/palate, movement disabilities, chronic respiratory diseases, chronic skin diseases, and thyroid diseases. The questionnaire included a consent statement declaring each participant’s voluntary agreement to participate in this study. In this part of the questionnaire, the researcher clarified that all collected data would be handled confidentially and utilised for research purposes only.

### 2.3. Statistical Analysis

Statistical analysis was performed using Statistical Package for the Social Sciences^®^ (SPSS) software (version 20.0, IBM Corp., Armonk, NY, USA). Categorical variables were summarised as frequencies and percentages. The distribution of continuous variables was analysed using means ± standard deviation (SD). The association between categorical variables was investigated and reported using the chi-squared test and associated *p*-values. In statistical tests, *p*-values < 0.05 were considered significant.

## 3. Results

### 3.1. Participant Characteristics

This study included a total of 1010 participants who were willing to complete the online-distributed questionnaire. Of these, 56% were female, and 57% were aged ≥31 years. The percentage of participants with an undergraduate qualification was 68%. The majority of the participants were employed (57%) and were married or in a relation at some point in their lives (*N* = 757; 75%). A large proportion of the participants reported an income <10 k SAR per month (Table 1). The Albaha region consists of 10 provinces, and the citizens of Albaha province comprised 62% of the participants. The average age at marriage was estimated to be 24 ± 5 years.

### 3.2. Prevalence of Consanguinity among Albaha Citizens

**Figure 1:**
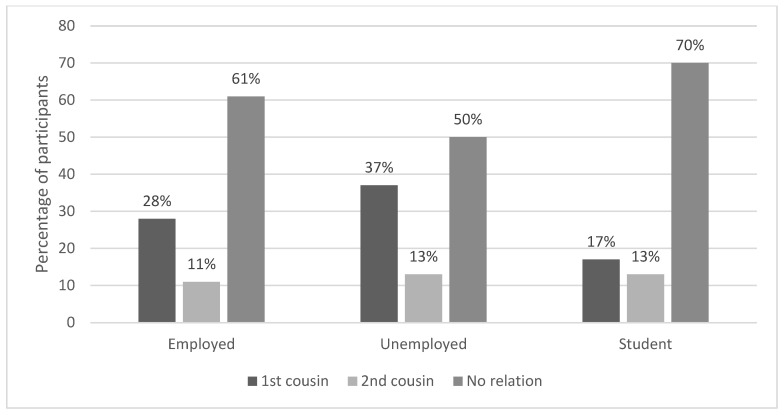
Comparison of prevalence of CM among Saudi citizens in Albaha according to employment status.

Of the total married participants, 302 (40%) were in a CM, and of these, 71.5% and 28.5% were married to first or second cousins, respectively. Age, education level, and monthly income showed no significant influence on the rate of CM among Saudi citizens in Albaha. The rate of CM among students (mean age ± SD, 23 ± 6 years; 12% earning more than 5 k SAR per month) was 30%, which was significantly lower than the employed participants (mean age ± SD, 40 ± 10 years; 89% earning more than 5 k SAR per month) and unemployed participants (mean age ± SD, 34 ± 11 years; 43% earning more than 5 k SAR per month) (*p* = 0.0098) (Table 2 and Figure 1).

**Figure 2:**
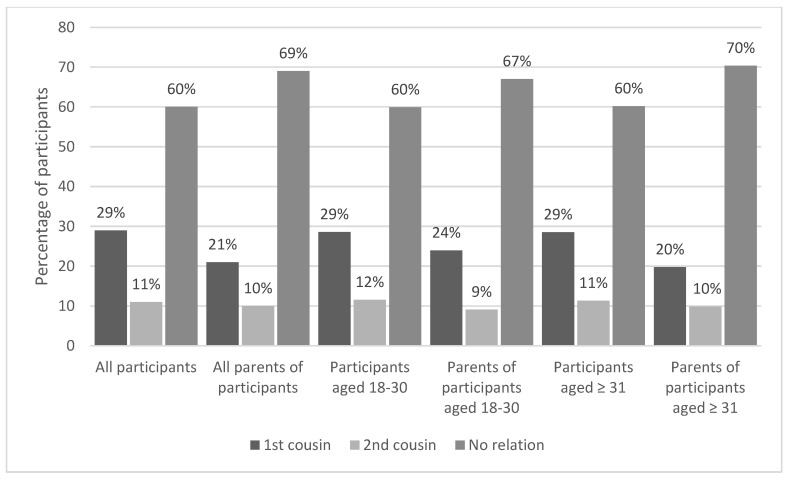
Comparison of prevalence of CM according to the ages of the participants and those of their parents.

Comparison of the prevalence of CM between two generations, the participants and their parents, showed that the parents of the participants had a lower proportion of CM compared to the participants themselves (31% vs. 40%), and the rate of first-cousin marriage remained higher among the participants than their parents (Figure 2). Association analysis indicates that CM among the parents of the participants is significantly associated with a higher rate of CM among the participants themselves (*p* < 0.001), with 60% of participants with parents in a CM were also in a CM, whilst 32% of participants with unrelated parents were in a CM.

### 3.3. Incidence of Hereditary Diseases and Health-Related Issues Associated with CM

Of the pre-selected screening panel for hereditary diseases and health-related issues among participants, which involved 17 items, CVDs (*p* < 0.001), blood diseases (*p* < 0.001), cancer (*p* = 0.046), hearing loss and speech disorders (*p* = 0.003), and ophthalmic diseases (*p* = 0.037) were significantly associated with CM. Of the participants’ children, 92% of those with CVDs were found to have related parents. The percentage of occurrence of blood diseases among children of related parents was 3 times higher than that among children of unrelated parents (79% and 21%, respectively). Cancer was reported in 2% of the children of related parents and in 0.66% of the children of unrelated parents. Issues related to speech and hearing ability were reported in 98% of the children of related parents, as compared to 0.22% of the children of unrelated parents. Children of unrelated parents showed a lower rate of ophthalmic diseases than children of related parents (Table 3).

## 4. Discussion

Consanguinity has been practiced by humans since the earliest days, and it remains a common mode of marriage among Arabs and in Muslim countries. To the best of our knowledge, this study is the first to explore the prevalence of CM and associated hereditary diseases among Saudi citizens in Albaha. The current study involved 757 participants who were married, divorced, or widowed, and it revealed that 40% (*N* = 302) were in a CM, of which 216 (71.5%) married their first cousins. Although the prevalence of CM among Saudi citizens in Albaha was high, it remains near the lower estimate compared to other cities in Saudi Arabia and several other Gulf countries, where it ranges between 40% and 60% [2,3,4,5,6,7,8]. Furthermore, the current prevalence of CM among Saudi citizens in Albaha remains close to the prevalence estimated in 2005 (42.1%) in a study by El-Mouzan, et al. (2007), which estimated the prevalence of CM in different cities in Saudi Arabia. This previous study estimated the difference between rural and urban provinces in Albaha in terms of the prevalence of CM, which were 45% and 34%, respectively [6]. However, in our study, we were unable to observe such a variation between rural and urban provinces, which might be due to the more homogenous nature of urbanisation among the Albaha provinces, which is believed to be a result of the promotion of quality of life and services in the different provinces of Albaha, in line with the guidelines of *Saudi Vision 2030.* The prevalence of CM among participants was higher than that among their parents (40% versus 31%, respectively). This finding requires further investigation as it might be an indicator of an increase in the CM over time. Several factors may influence the trend estimate, such as the migration of citizens into or away from Albaha due to people looking for an area with lower living costs, better quality of life and services, or better job opportunities. Our investigation indicates that education and income levels had no significant influence on the frequency of CM among Saudi citizens in Albaha, which does not concur with the evidence in the literature. Studies that explored the association between economic status, education level, and CM frequency have reported a reverse association between these two factors and CM [11,19]. Unemployment showed a significant association with a higher frequency of CM; this association could not be justified by the variation in age or monthly income of the participants. Significantly, the prevalence of CM among the participants was associated with the CM status of participants’ parents.

The practice of CM among Arabs seems to have originated from the need for early urban and rural societies to maintain or increase the number of individuals within small or limited communities, enter into alliances with communities surrounding a geographical area, and ensure that both partners shared a similar ethnic background, or cultural, sect, or religious identities. Nowadays, the practice of CM among Arabs may exist because of the intention to preserve family heritage, whether it is owing to cultural, financial, or geographical reasons, or for strengthening family ties. Among the study population, CM seems to be mainly practiced for cultural reasons and to strengthen family ties, which has been reported previously among many neighbouring populations with Arab and Muslim backgrounds [1,8]. It is worth noting that social changes associated with urbanisation have failed to positively affect the practices of CM among Arabs and the Muslim population [20,21,22]. 

Practising CM is not an issue by itself; however, the health issues and hereditary diseases which occur because of this type of marriage could negatively affect the well-being of future generations and, consequently, affect healthcare systems. NCDs are found to cause premature death, which comprises 73% of all deaths among Saudi citizens. On the other hand, diseases caused by inherited genetic disorders, such as CVDs and cancer, account for approximately 50% of all cases of NCDs [23]. Evidence for the association between the incidence of hereditary diseases and CM is well-established. CM has been found to be related to increased risks of haemoglobinopathies, intellectual disabilities, congenital glaucoma, ciliopathies, disorders related to inborn errors of metabolism, retinal dystrophies, hearing loss, primary microcephaly, and familial hypercholesterolaemia [24,25,26,27]. Such health issues and diseases resulting from CM can negatively impact the economy and the healthcare system [15,28]. Given that the practice of CM among the Saudi population has maintained a high prevalence among different cities in Saudi Arabia over time, and clear evidence associating this type of marriage with several hereditary diseases and health issues, there is an urgent need for immediate interventions. These may involve the application of a pre-marriage screening program for hereditary diseases and other health issues known to be associated with CM, as well as the implementation of a proper educational program to raise the awareness of the targeted population regarding the negative consequences of CM. Evidence reported in the literature suggests that educational programs on the consequences of CM or consultation sessions based on outcomes from pre-marriage screening programs for hereditary diseases have managed to successfully alter culturally rooted behaviours, positively influence the well-being of the population, and aid in the efficient use of healthcare resources [1,29,30,31,32,33,34].

This study has a few limitations. The snowball technique was used to distribute the questionnaire among Saudi citizens in Albaha; thus, there was a lack of evidence to estimate the response rate. In addition, there was a chance of including duplicated information when two partners from one relationship submitted their information. These issues can be avoided in future work by utilising a different method for sampling the population. Regarding the questionnaire design, the author avoided any questions that would make participants uncomfortable in completing the questionnaire; such questions included those which would solicit private information or personal decisions or preferences (i.e., What is the reason for choosing to engage in CM?). For a better understanding of CM, the latter type of question may be considered in a study of CM among other populations with different cultural backgrounds.

## 5. Conclusions

This study indicated a high prevalence of CM among Saudi citizens in Albaha, with a higher proportion of marriages among first cousins compared to those among second cousins. In addition, a comparison of different generations revealed that recent generations have a higher prevalence of CM than previous generations. Moreover, the analysis suggested an association between the incidence of hereditary diseases and health-related issues and CM. Further work needs to establish trends of CM over time among populations expected to have high rates, as well as to improve the productivity of individuals from the coming generations and efficiently use healthcare resources. The current national pre-marriage screening program should be extended to involve screening for genetic disorders that lead to hereditary diseases among offspring from CMs.

## Figures and Tables

**Table 1:** Sociodemographic characteristics of the participants.

		Frequencies		Age	Monthly Income (SAR) *									
		N	%	Mean ± SD	<5 k		>5 k		>10 k		>15 k		>25 k	
All (*N* = 1010)					N (%)		N (%)		N (%)		N (%)		N (%)	
Age (years)														
	18–38	639	63	26 ± 6	356 (56)		120 (19)		104 (16)		31 (5)		28 (4)	
	≥39	371	37	47 ± 7	42 (11)		84 (23)		134 (36)		86 (23)		25 (7)	
Gender														
	Female	563	56	39 ± 10	157 (35)		107(24)		114 (26)		47 (11)		22 (5)	
	Male	447	44	31 ± 12	241 (43)		97 (17)		124 (22)		70 (12)		31 (6)	
Education level														
	General education *	209	21	35 ± 15	117 (56)		42 (20)		37 (18)		6 (3)		7 (3)	
	Undergraduate education	690	68	34 ± 11	268 (39)		150 (22)		156 (23)		84 (12)		32 (5)	
	Graduate education	111	11	37 ± 8	13 (12)		12 (11)		45 (41)		27 (24)		14 (13)	
Work status														
	Employed	573	57	40 ± 10	60 (11)		161 (28)		213 (37)		109 (19)		30 (5)	
	Unemployed	150	15	34 ± 11	85 (57)		28 (19)		17 (11)		3 (2)		17 (11)	
	Student	287	28	23 ± 6	253 (88)		15 (5)		8 (3)		5 (2)		6 (2)	
Provinces														
	Albaha	627	62	36 ± 12	225 (36)		127 (20)		154 (25)		88 (14)		33 (5)	
	Alhajrah	13	1	30 ± 8	4 (31)		1 (8)		7 (54)		1 (8)		0 (0)	
	Alaqeeq	46	5	30 ± 11	26 (57)		8 (17)		7 (15)		3 (7)		2 (4)	
	Alqura	52	5	31 ± 12	25 (48)		7 (14)		11 (21)		6 (12)		3 (6)	
	Almekhwah	37	4	32 ± 11	15 (41)		12 (32)		5 (14)		4 (11)		1 (3)	
	Almandaq	65	6	34 ± 10	22 (34)		15 (23)		21 (32)		3 (7)		4 (6)	
	Baljurashi	64	6	31 ± 12	25 (39)		11 (17)		16 (25)		7 (11)		5 (8)	
	Bani-hassan	49	5	30 ± 12	27 (55)		10 (20)		7(14)		4 (8)		1 (2)	
	Ghamed Alzenad	29	3	34 ± 11	12 (41)		8 (28)		6 (21)		0 (0)		3 (10)	
	Qelwah	28	3	32 ± 14	17 (61)		5 (18)		4 (14)		1 (4)		1 (4)	
Marital status														
	Single	253	25	21 ± 3	227 (90)		17 (7)		2 (1)		5 (2)		2 (1)	
	Widow	17	2	43 ± 14	8 (47)		1 (6)		4 (24)		2 (12)		2 (12)	
	Married	709	70	39 ± 10	150 (21)		180 (25)		225 (32)		106 (15)		48 (7)	
	Divorced	31	3	33 ± 11	13 (42)		6 (19)		7 (23)		4 (13)		1 (3)	
Monthly income (SAR) *														
	<5 k	204	20	26 ± 9	-	-	-	-	-	-	-	-	-	-
	>5 k	398	39	37 ± 10	-	-	-	-	-	-	-	-	-	-
	>10 k	238	24	41 ± 10	-	-	-	-	-	-	-	-	-	-
	>15 k	117	12	42 ± 10	-	-	-	-	-	-	-	-	-	-
	>25 k	53	5	39 ± 14	-	-	-	-	-	-	-	-	-	-

* General education involves participants with primary, secondary, or higher education. SAR: Saudi Arabian riyals.

**Table 2:** Prevalence of CM among the participants.

		Consanguineous Marriage						Nonconsanguineous Marriage		*p*-Value ^†^
		1st Cousin		2nd Cousin		Total *				
		N	%	N	%	N	%	N	%	
All (*N* = 757)		216	72	86	28	302	40	455	60	-
Age (years)										
	18–38	118	77	35	23	153	39	235	61	0.790
	≥39	98	66	51	34	149	40	220	60	
Education level										
	General education	39	68	18	32	57	39	89	61	0.962
	Undergraduate education	144	72	57	28	201	40	302	60	
	Graduate education	33	75	11	25	44	41	64	59	
Work status										
	Employed	154	72	59	28	213	39	337	61	0.0098
	Unemployed	49	74	17	26	66	50	65	50	
	Student	13	57	10	43	23	30	53	70	
Monthly income (SAR)										
	<5 k	49	72	19	28	68	40	103	60	0.696
	>5 k	67	87	10	13	77	41	110	59	
	>10 k	57	58	42	42	99	42	137	58	
	>15 k	28	74	10	26	38	34	74	66	
	>25 k	15	75	5	25	20	39	31	61	

* Indicates the total number and the percentage of participants with a first- or second-cousin marriage. † *p*-values were estimated using a chi-squared test for comparison between two groups of participants: those in a consanguineous marriage and those in a nonconsanguineous marriage. SAR: Saudi Arabian riyals.

**Table 3:** Prevalence of health-related issues and hereditary diseases among the participants’ children according to consanguinity.

		Consanguineous Marriage						Nonconsanguineous Marriage		*p*-Value ^†^
		1st Cousin		2nd Cousin		Total *				
		N	%	N	%	N	%	N	%	
Blood pressure										
	Yes	7	44	1	6	8	50	8	50	0.372
	No	206	28	85	12	218	40	447	60	
Diabetes										
	Yes	19	40	6	13	25	52	23	48	0.172
	No	197	28	80	11	277	49	432	61	
Cardiovascular diseases										
	Yes	11	85	1	8	12	92	1	8	<0.001
	No	205	28	85	11	290	49	454	61	
Blood diseases										
	Yes	19	66	4	14	23	79	6	21	<0.001
	No	197	27	82	11	279	32	449	62	
Obesity										
	Yes	8	36	0	0	8	36	14	64	0.211
	No	208	28	86	12	294	40	441	60	
Cancer										
	Yes	6	67	0	0	6	67	3	33	0.046
	No	210	28	86	12	296	40	452	60	
Down syndrome										
	Yes	1	17	1	17	2	34	4	66	0.091
	No	215	29	85	11	300	40	751	60	
Chronic gastrointestinal disorders										
	Yes	3	38	0	0	3	38	5	63	0.562
	No	213	28	86	12	299	40	450	60	
Mental disorders										
	Yes	1	17	0	0	1	17	5	83	0.168
	No	215	29	86	12	301	40	450	60	
Hearing loss and speech disorders										
	Yes	6	86	0	0	6	86	1	14	0.003
	No	210	28	86	12	296	49	454	61	
Rheumatoid arthritis										
	Yes	0	0	0	0	0	0	2	100	0.514
	No	216	29	86	11	302	40	453	60	
Ophthalmic diseases										
	Yes	25	33	14	19	39	52	36	48	0.037
	No	191	28	72	11	263	49	419	61	
Cleft lip and palate										
	Yes	2	67	0	0	2	67	1	33	0.327
	No	214	28	86	12	300	40	454	60	
Movement disabilities										
	Yes	0	0	1	50	1	50	1	50	0.198
	No	216	29	85	11	301	40	454	60	
Chronic respiratory diseases										
	Yes	7	28	4	16	11	44	14	56	0.754
	No	209	29	82	11	291	40	441	60	
Chronic skin diseases										
	Yes	4	44	2	22	6	66	3	34	0.242
	No	212	28	84	11	296	40	452	60	
Thyroid disease										
	Yes	0	0	0	0	0	0	0	0	-
	No	216	29	86	11	302	40	455	60	

† *p*-values were estimated using the chi-squared test for comparison between two groups of participants, consanguineous marriage and nonconsanguineous marriage. * Indicates total number and percentage of participants having 1st Cousin or 2nd Cousin relationship.

## Data Availability

The datasets used and/or analysed in the current study are available from the corresponding author upon reasonable request.
